# Scientific Transparency: a New Horizon for Scientific Publication and the “New Cardiovascular Surgeon”

**DOI:** 10.21470/1678-9741-2019-0609

**Published:** 2019

**Authors:** Domingo M. Braile, Meryt Zanini, Camila Sáfadi Alves Gonçalves, Paulo Roberto B. Evora

**Affiliations:** 2 Faculdade de Medicina de São José do Rio Preto (FAMERP), São José do Rio Preto, SP, Brazil; 3 Universidade de Campinas (UNICAMP), Campinas, SP, Brazil; 4 Postgraduate degree in Health Law from Faculdade de Saúde Pública da Universidade de São Paulo (USP), São Paulo, SP, Brazil; 5 MBA in Health Management from Fundação Getúlio Vargas (FGV), São Paulo, SP, Brazil; 6 Sociedade Brasileira de Cirurgia Cardiovascular, São Paulo, SP, Brazil; 8 Faculdade de Medicina de Ribeirão Preto da Universidade de São Paulo (FMRP-USP), Ribeirão Preto, SP, Brazil

The term ‘innovation’ in cardiovascular surgery is not new: there are several publications in national and international journals that show the field’s rich tradition in the development of surgical techniques and medicines, among other products. However, in recent years, information technology has significantly impacted medical practice, and with this, several challenges have emerged, such as the provision and dissemination of health care information.

With the constant growth of health research, falsification, plagiarism, and data fabrication have been having a deleterious effect on the results presented. Unethical conduct in research drains the foundations of science which, by their very nature, are built on previous evidences, and if these are false, these foundations are logically weakened. In this context, it is up to the hard work of the editors of major scientific publications to ensure to the researchers and the society the dissemination of papers with reliable and transparent results.

Research misconduct means fabrication, falsification, or plagiarism in proposing, performing, or reviewing research, or in reporting research results^[1]^. It is important to point out that misconduct does not include honest mistakes or differences of opinion.

Scientific integrity consists in sticking to strategies that allow the promotion and development of ethical and honest scientific standards that guarantee objectivity, clarity, reproducibility, openness, and accessibility, the caring duty, fairness in providing references and giving credit, and responsibility to future scientists and researchers.

This is one of the main concerns that scientific editors face daily, and this topic has been included in the Brazilian Journal of Cardiovascular Surgery’s (BJCVS) new instructions.

Plagiarism consists in the appropriation of someone else’s ideas, processes, results, or words, without giving the appropriate credits. The authors are responsible for the content and the information in their manuscripts. BJCVS uses the Similarity Check software, which allows to detect similarities in the submitted materials. The manuscripts that have been plagiarized will be rejected, and their authors may incur penalties determined by the journal’s Editorial Board.

Fraud consists in inventing data or results and falsifying and manipulating materials, equipment, or research processes, or in altering or omitting data or results, so that the research is not accurately represented in the research record. Fabrication is inventing data or results.

In 2012, a search in the PubMed database found 2,047 retractions between biomedical and life sciences research articles dating back to 1973^[2]^. More than two-thirds of these articles were retracted for misconduct in the research, mainly fraud or suspected fraud and publication duplication. In the course of the BJCVS’ processing, if fraud and/or data fabrication is identified, the manuscript will be summarily excluded from the evaluation process and its authors may incur penalties determined by the BJCVS’ Editorial Board.

Another concern is the redundant publication, which consists of the inadequate division of the study’s results into more than one article (a practice also known as “salami publication”). To assist editors, researchers, and readers in general, the BJCVS is based on the five criteria that determine a publication redundant^[3]^: (1) similar sample numbers and sizes; (2) identical or almost identical methodology; (3) similar results; (4) at least one author’s name is common to both reports; and (5) little or no new information is made available.

These are issues that have long been widely discussed by the BJCVS’ Editorial Board, and with the implementation and practice of well-defined policies and actions, we ensure ethical and transparent publications.

In addition to the very important information described with great emphasis in this Editorial, we also want to share with our authors and readers a situation that has been a source of uncertainty and often criticism.

It is the extension of the Cardiovascular Surgeon’s proper activities regarding the procedures for treatment of the heart and/or great vessels’ diseases.

With the advent of new technologies, mainly the less invasive ones, which are performed through catheters and percutaneous introducers, a large part of the surgical treatments has been taken over by different professionals, leaving the Cardiovascular Surgeons out of many techniques, once exclusive of their specialty.

Thus, our field has been restricted, since we have been denied the right to assume the new techniques on equal terms.

This is a restriction, which we consider improper and unethical, because the professional who had always performed surgical (invasive) procedures on the heart and great vessels would be prevented from treating the disorders of these organs, which were always an integral part of his/her portfolio of action, just because its way of approach has been modified.

Logically assuming this new posture, Cardiovascular Surgeons should be prepared to acquire proficiency in new technologies, attending compulsory courses, with rigorous assessments, demonstrating to be able to perform these innovations.

This process is in full swing at the Brazilian Society of Cardiovascular Surgery - SBCCV, with great success, following the universal trend.

The Residence, now lasting five basic years, and two more years for mastering the different areas of expertise, is one of the longest preparation periods to form what we have denominated the ‘New Cardiovascular Surgeon’^[4]^.

This professional will be an expert who will continue to be able to perform conventional surgical procedures, when these were indicated, or use new technologies, when preferred.

In China, that presents itself as a country where everything is happening quickly and efficiently, this new professional has already been acting for a long time, in accordance with this new attitude.

In 1967, therefore, for over 50 years, Chinese surgeons were already using transventricular procedures, utilizing introducers for closure of interventricular communication (IVC)^[5]^.

In the BJCVS^[6,7]^ [www.bjcvs.org], you can read two articles, also from China, in which the authors demonstrate closure of interatrial communications (atrial septal defects) (ASD)^[6]^ with occluders compared to the conventional closure, encouraging surgeons to use the minimally invasive technique.

The other article^[7]^, from 2019, compares the transventricular surgical procedure, with the percutaneous procedure for closure of Isolated Ventricular Septal Defect (VSD)^[7]^, proving that the first procedure (transventricular) is most efficient regarding time, absence of radiation, and safety.

I hope that, in Brazil, Cardiovascular Surgeons can treat all cardiovascular diseases with the method that is most suitable for each patient, being them able to perform the full range of procedures - invasive, minimally invasive, or transcutaneous -, Applied the best evidence and performed the state-of-the-art procedure. 
